# Progressive Acceleration of Insulin Exposure Over 7 Days of Infusion Set Wear

**DOI:** 10.1089/dia.2022.0323

**Published:** 2023-01-27

**Authors:** Jasmin R. Kastner, Timothy S. Bailey, Poul Strange, Leon Shi, Keith A. Oberg, Paul J. Strasma, Jeffrey I. Joseph, Douglas B. Muchmore

**Affiliations:** ^1^ The Artificial Pancreas Center, Department of Anesthesiology, Thomas Jefferson University, Philadelphia, Pennsylvania, USA.; ^2^ Capillary Biomedical, Inc., Irvine, California, USA.; ^3^ AMCR Institute, Escondido, California, USA.; ^4^ Integrated Medical Development, Princeton Junction, New Jersey, USA.; ^5^ Orthogonal Concept Consulting, Valencia, California, USA.

**Keywords:** Extended-wear insulin infusion sets, Type 1 diabetes, Euglycemic clamp, Insulin pharmacokinetics

## Abstract

Insulin exposure varies over 3 days of insulin infusion set (IIS) wear making day-to-day insulin dosing challenging for people with diabetes (PWD). Here we report insulin pharmacodynamic (PD) and pharmacokinetic (PK) data extending these observations to 7 days of IIS wear. PWD (A1C ≤8.5%, C-peptide <0.6 nmol/L, ≥6 months pump use) were enrolled in a crossover euglycemic clamp pilot study comparing conventional Teflon angled IISs with an investigational extended-wear IIS. PK/PD data from six participants were obtained for 5 h postbolus. Although PD data were unstable, PK profiles (pooled data from both groups) of insulin lispro (0.15 U/kg bolus) showed statistically significant progressive decreases from days 0 to 7 for *t*_max_ (*P* < 0.001), *C*_max_ (*P* < 0.05), and mean residence time (*P* < 0.0001). Area under the insulin concentration curve (AUC_0–300_) declined by ∼24% from days 0 to 7 (*P* < 0.05). These results confirm/extend previous observations showing progressive acceleration of insulin exposure over IIS wear time. This may have implications for PWD and designers of closed-loop algorithms, although larger studies are necessary to confirm this. The study was registered in clinicaltrials.gov (NCT04398030).

## Background

The variability of subcutaneously (SC) administered insulin absorption, even within the same person and the same insulin infusion set (IIS), presents a significant challenge for insulin pump users.^1–4^ Little data are available on the change in insulin pharmacokinetics (PK) and pharmacodynamics (PD) beyond 3 days of IIS wear. Available data have shown acceleration of insulin absorption over time.^5,6^

In this study, we present PK/PD data generated from a proof-of-concept clamp study showing a very consistent change from immediately after insertion up to a full 7 days of IIS wear. We compared a commercially available angled 3-day Teflon IIS with an extended-wear IIS prototype and hypothesized that the area under the glucose infusion rate curve [AUC_(GIR)_]—and, therefore, the glucose lowering effect of insulin—would remain stable over 7 days of wear using the novel IIS.

## Methods

The Capillary Biomedical, Inc. investigational extended-wear (CBX) IIS is a sterile single-use device for continuous SC insulin infusion. It contains a coil-reinforced soft polymer (Nylon-derivative) indwelling cannula with one distal and three proximal holes. CBX IISs are designed to be used with commercially available infusion pumps for an extended period (i.e., 7 days).

We performed a prospectively enrolled randomized sequence two-way crossover study of the investigational CBX (Capillary Biomedical, Inc., Irvine, CA) and a commercial angled Teflon (MiniMed™ Silhouette™; Medtronic Diabetes Care, Northridge, CA; indicated for 3-day use) IIS for two weeklong home use periods with four in-patient euglycemic clamp sessions during each of the 7-day periods. Between the two periods, there was a wash out of 14 days (Supplementary Fig. S1).

WCG IRB approved the protocol and all associated documents used by participants before initiation of the study (Study No. 1283383) and during the study were registered in clinicaltrials.gov (NCT04398030). Participants aged 18–70 years, diagnosed with type 1 diabetes mellitus, and using rapid-acting insulin analog delivered through a Medtronic MiniMed insulin pump (model 530 or higher; Medtronic Diabetes Care) for at least 6 months were enrolled.

Euglycemic clamp experiments were performed at AMCR Institute (in Escondido, CA.) on days 0, 3, 5, and 7 in each treatment period (eight clamps per participant). Participants were admitted to AMCR the evening before the clamp study and glucose was stabilized overnight with intravenous (IV) insulin or glucose as needed. Personal pumps were set to 0.1 U/h overnight and throughout the clamp experiment. Once glucose was stable (95 ± 15 mg/dL) in the morning, IV insulin was stopped 20 min before administering a bolus of 0.15 U/kg of insulin lispro (Eli Lilly, Indianapolis, IN) by pump. Glucose was clamped at 95 ± 15 mg/dL for 300 min or terminated early if runaway hyperglycemia occurred.

Per participant and clamp, 30 plasma samples were collected in regular intervals and stored at −70°C. Samples were analyzed elsewhere (Northern Lights Mercodia Lispro ELISA, Mercodia AB, Uppsala, Sweden; lowest limit of quantification (LLOQ) = 1.0 mU/L, 94% accuracy^7^). Owing to the small sample size and the intrinsically greater variability in pharmacodynamic measures, the PK results are the only results detailed in this report.

PK parameters (area under the insulin concentration curve [AUC] over the first 60 or 300 min, respectively; time to maximum insulin concentration [*t*_max_]; maximum insulin concentration [*C*_max_]; time to half-maximum insulin concentration, early and late [*t*_50%_], and mean residence time [MRT]) were tested for treatment effects. Parameters were log transformed as data distribution was found to be skewed. Estimated means were then back transformed and the ratio between treatments/days (equality means a ratio of 1) tested for significance using mixed model for repeated measures (two-sided, *α* = 0.05). For simplicity reasons, all data are presented as median (25th–75th percentiles) or mean ± standard deviation.

## Results

An initial sample size calculation determined *n* = 24 to generate 90% power for a comparison of changes in glucose infusion rate (GIR) over time. The study was terminated early due to difficulties executing clamp procedure as planned. Specifically, the lag time between manual glucose testing and subsequent GIR adjustment proved more difficult than anticipated. Of 19 screened participants, 12 participants failed screening and 7 participants (average [ ± SD] age 40.2 ± 9.2 years, diabetes duration 23.5 ± 16.7 years, and body mass index 27.6 ± 16.7 kg/m^2^, two females) were enrolled. During Control IIS use, 5 participants completed 4 clamps, and 1 participant completed 3 clamps of which 2 clamps were used for analysis (22 total).

One participant completed 1 clamp in the Control group but none in the CBX group and this data set was excluded from the analysis. In the CBX group, 5 participants completed 4 clamps, and 1 participant completed 3 clamps (23 total). Ultimately, data from 6 participants (per-protocol population) and 45 clamp procedures were used for the analysis. Multiple comparisons of PD parameters between days were not done due to unstable baseline glucose (Supplementary Fig. S2), making it difficult to execute clamp procedures efficiently (GIR curves shown in Supplementary Fig. S3) and resulting in nonrobust PD data (Table 1).

**Table 1.** Median Key Pharmacokinetic and Pharmacodynamic Parameters with Interquartile Range

Variable	Day 0, median (25th–75th)		Day 3, median (25th–75th)		Day 5, median (25th–75th)		Day 7, median (25th–75th)		p, day 7 vs. 0		
IIS type	CBX	Control	CBX	Control	CBX	Control	CBX	Control	CBX	Control	Pool
n (PP)	6	6	6	6	6	6	5	5			
Pharmacokinetics											
*t*_max_ (min)	65.0 (50.0–90.0)	67.5 (45.0–80.0)	42.5 (35.0–45.0)	32.5 (25.0–50.0)	25.0 (25.0–30.0)	25.0 (25.0–30.0)	25.0 (25.0–25.0)	25.0 (20.0–30.0)	<0.001	0.001	<0.0001
*C*_max_ (mU/L)	60.4 (33.5–73.6)	61.9 (45.0–82.5)	69.0 (56.2–71.5)	60.2 (56.2–69.7)	93.6 (73.9–104.4)	72.6 (54.1–91.0)	92.3 (78.4–101.5)	80.9 (79.0–84.0)	0.11	0.22	0.049
*t*_50% (early)_ (min)	23.3 (11.8–34.1)	27.4 (19.8–32.7)	18.0 (11.2–19.2)	15.7 (12.2–25.6)	12.4 (10.3–14.2)	11.5 (8.2–13.1)	11.6 (9.5–14.2)	12.3 (11.5–16.4)	0.002	0.0001	<0.0001
*t*_50% (late)_ (min)	163.1 (157.3–181.8)	156.5 (121.2–174.5)	135.4 (120.4–150.0)	119.3 (98.8–152.7)	74.8 (66.7–76.2)	69.3 (68.2–88.3)	62.5 (60.7–68.8)	63.5 (57.5–77.9)	<0.0001	<0.0001	<0.0001
MRT (min)	129.5 (117.1–137.5)	118.0 (97.9–131.0)	114.4 (105.9–116.9)	112.4 (104.1–124.3)	89.0 (76.9–91.7)	88.7 (85.7–110.5)	75.7 (66.7–87.0)	75.4 (62.2–98.6)	<0.0001	<0.0001	<0.0001
AUC_0–60_ mU/(min·L)	2294 (1139–2757)	2264 (1732–2806)	2604 (2324–3511)	2410 (1666–2686)	4068 (3148–4364)	2972 (1704–3915)	3640 (3636–4329)	3034 (3009–3680)	0.05	0.07	0.009
AUC_0–300_ mU/(min·L)	8562 (6576–11416)	8894 (7599–9870)	8281 (7176–10737)	7434 (6600–8282)	8175 (7194–8538)	7253 (5949–7625)	6684 (6402–8342)	6603 (5822–6773)	0.11	0.09	0.023
Pharmacodynamics											
*t*_max(GIR)_ (min)	140.0 (80.0–170.0)	145.0 (90.0–190.0)	65.00 (60.0–120.0)	82.0 (60.0–110.0)	62.5 (50.0–70.0)	59.0 (55.0–60.0)	55.0 (50.0–55.0)	70.0 (60.0–70.0)	Not done due to large variability and difficulty executing clamp procedure.		
GIR_max_ [mg/(kg·min)]	8.4 (4.5–9.1)	10.2 (7.7–11.7)	7.41 (5.7–9.5)	5.2 (5.0–13.5)	8.1 (6.5–10.9)	9.1 (7.3–10.8)	10.1 (4.9–11.3)	9.2 (6.9–11.5)			
Onset of action (min)	28.9 (21.4–39.0)	76.1 (56.6–106.4)	28.2 (12.1–34.0)	35.4 (29.0–41.4)	21.1 (18.4–23.8)	20.0 (16.4–23.0)	18.8 (16.2–27.0)	27.5 (14.6–32.0)			
*t*_50% (GIR, early)_	48.7 (25.7–63.5)	67.6 (43.0–93.5)	49.0 (37.7–95.0)	51.3 (44.9–55.6)	33.3 (28.7–37.0)	38.8 (30.6–39.1)	32.2 (28.0–36.0)	48.1 (23.2–48.1)			
*t*_50% (GIR, late)_	187.4 (160.4–234.3)	182.6 (164.3–247.3)	157.6 (97.5–179.0)	147.8 (88.3–177.7)	92.6 (77.1–147.0)	106.8 (95.4–140.2)	107.5 (105.9–117.8)	96.7 (87.1–109.5)			
AUC_0–300(GIR)_ (mg/kg)	1205 (642.1–1219)	1330 (790.6–1539)	719.6 (652.3–864.3)	743.1 (585.5–1268)	663.5 (609.8–1332)	695.2 (585.4–1395)	883.0 (380.6–908.4)	687.7 (386.7–1039)			

The ratios of estimated means were compared between day 0 (day of insertion, *n* = 6) and day 7 (day of removal, *n* = 5) using *Mixed Models for Repeated Measures* for statistical significance testing. For simplicity, the medians and 25th to 75th percentile are presented here. There was no statistically significant difference between pharmacokinetic parameters of each group (CBX vs. Control on days 0 and 7, respectively). The last column shows statistical comparison of day 7 and day of insertion pooling the data from both IIS types.

AUC_0–*t*_, area under the insulin concentration curve from 0 min until specified time point; *C*_max_, maximum insulin concentration; CBX, CapBio Extended, Wear; GIR_max_, maximum glucose infusion rate; IIS, insulin infusion set; MRT, mean residence time; PP, per-protocol; *t*_max_, time to maximum insulin concentration; *t*_50% (early)_, time to reach 50% of *C*_max_ before peak; *t*_50% (late)_, time to reach 50% of *C*_max_ after peak; *t*_max(GIR)_, time to reach GIR_max_; *t*_50% (GIR, early)_, time to reach 50% GIR_max_ before peak; *t*_50% (GIR, late)_, time to reach 50% GIR_max_ after peak; AUC_0–300(GIR)_, area under the GIR curve from 0 to 300 min.

Mean (±SD) time in tight glucose range (80–110 mg/dL) during clamp procedures was 85% ± 9% on day 0, 77% ± 15% on day 3, 80% ± 15% on day 5, and 70% ± 20% on day 7 averaged over all executed clamps. Average daily insulin dose and sensor glucose are shown in Supplementary Figure S4.

**FIG. 1.**
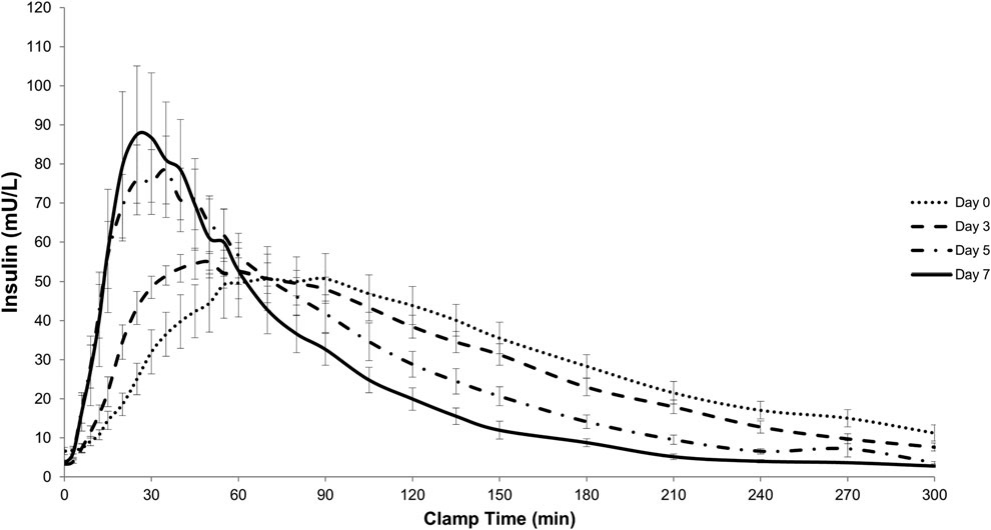
Insulin exposure over clamp time for combined data set (investigational CBX and Control infusion sets). Curves show average insulin concentration with Standard Error of the Mean. CBX, CapBio extended-wear.

We found statistically significant changes in PK parameters between day of IIS insertion (day 0) and day 7 of wear in each group (Fig. 1, Table 1, and Supplementary Fig. S5). However, there were no meaningful differences between IIS types for any of the parameters and data were pooled to highlight the significant changes in PK parameters over 7 days of IIS wear (last column in Table 1). By day 7, median time to peak insulin concentration (*t*_max_) was accelerated by 61% (from 65.0 to 25.0 min) in the CBX group (*P* ≤ 0.001) and by 63% (from 67.5 to 25.0 min) in the Control group (*P* = 0.001), and median early exposure (AUC_0–60_) increased by 59% (CBX, *P* = 0.05) and 34% (Control, *P* = 0.07), respectively.

Median MRT decreased over time by 42% in the CBX and 37% in the Control group, indicating significantly shorter insulin exposure by day 7 in both groups (both *P* < 0.0001). Median total insulin exposure (AUC_0–300_) showed a trend to decline by 22% (CBX, *n.s.*) and 26% (Control, *n.s.*), respectively, from days 0 to 7; when both infusion sets were analyzed together, this decline over 7 days was significant (*P* = 0.023). Peak insulin concentration (*C*_max_) increased by 36% in both groups over 1 week of IIS wear (*n.s.* individually but significant when combined, *P* = 0.049).

No severe or serious adverse events occurred during the study. A summary of all reported device-related events is given in Supplementary Table S1.

## Conclusions

Insulin absorption (PK) is known to show intrapatient variability on each of 3 days of IIS wear during insulin pump therapy.^1,4,6^ In this study, we extended PK observations to 7 days of IIS wear performing 4 euglycemic clamp experiments on days 0, 3, 5, and 7 of IIS wear after SC insulin bolus administration. Despite a small sample size, the PK data are robust, and showed a consistent and statistically significant progressive change in insulin bolus time–exposure profile parameters over 7 days of IIS wear. Owing to difficulties executing the clamp procedure and variable baseline glucose profiles, the PD data presented herein were not robust enough for extensive intra- and inter-IIS type statistical comparison.

We observed a reduction in overall insulin AUC during the 5 h postbolus but insulin absorption in the first hour significantly increased and accelerated as a function of IIS wear time. Insulin absorption from the tissue into the vasculature and lymphatics is influenced by various factors including SC blood flow, age of infusion site, location, and acute and ongoing inflammation.^1,6,8–11^ In the controlled in-patient setting in this study, marked differences in insulin exposure were observed over IIS wear time.

Interestingly, we found no difference in insulin PK/PD between the two IIS types. Although one IIS was designed for extended wear, we assume that the main factor for the ability to extend infusion set wear is the individual user and/or the permission to wear a 3-day IIS beyond its indication. This remains a topic to be further investigated.

Our PK findings confirm and extend previous observations of acceleration of insulin exposure in both humans and swine as infusion set wear time increases.^5,6,10^ Currently, more research is being conducted to develop extended-wear IISs to decrease patient burden caused by frequent site rotation, loss of infusion sites due to scarring, and increased cost.^5,12–15^ As insulin requirements change with extended IIS wear, information on the change in insulin PK is crucial to develop an optimal system for users.

It may be of importance to combine extended-wear IISs with automated insulin delivery (AID, artificial pancreas) to assist people with diabetes (PWD) with changes in bolus insulin dose and timing to maintain optimum glucose control. We have previously shown that conservative manual insulin dosing can lead to deteriorating glucose control and decreased time in range when using an IIS for longer than 3 days.^12^

In conclusion, this study confirms previous findings of significant changes in insulin absorption that we believe should be taken into consideration by PWD using IIS, those who care for them, and designers of AID systems.

## Supplementary Material

Supplemental data

Supplemental data

Supplemental data

Supplemental data

Supplemental data

Supplemental data
