# Supplementary material for: Progressive Acceleration of Insulin Exposure Over 7 Days of Infusion Set Wear
Source: Diabetes Technol Ther. 2023 Jan 27;25(2):143–7. doi: 10.1089/dia.2022.0323 (PMC9894594; doi:10.1089/dia.2022.0323)
Supplement: Supplemental data [file Supp_FigS1.docx]

**PROGRESSIVE ACCELERATION OF INSULIN EXPOSURE OVER SEVEN DAYS OF INFUSION SET WEAR - Supplementary Data**

**
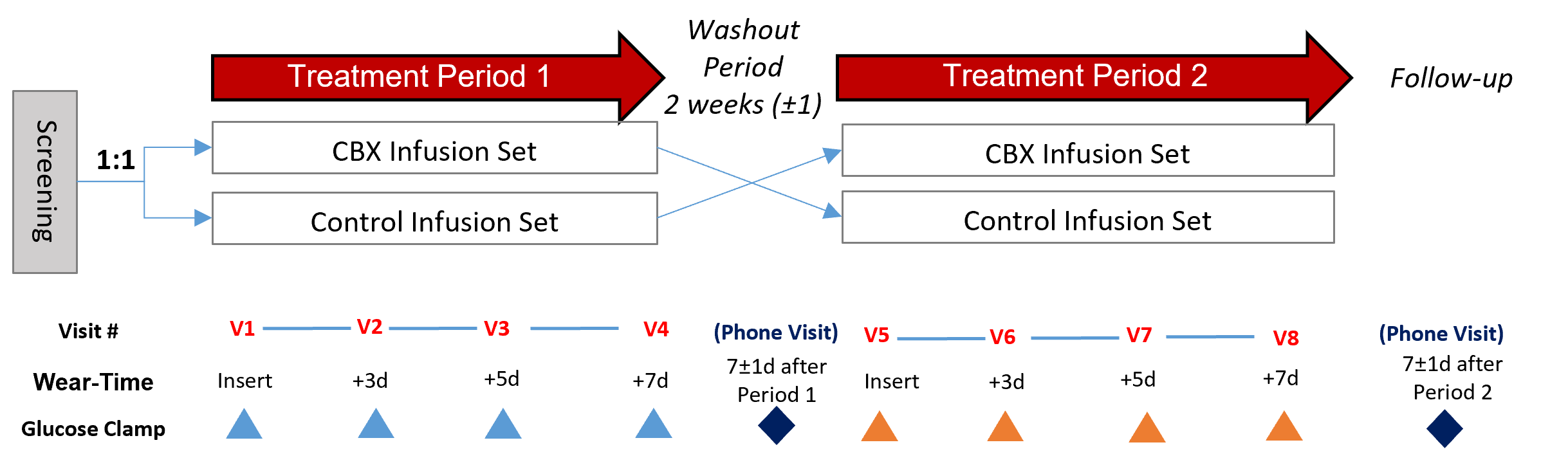
**

**Figure S1:** Study schematic.
