# Supplementary material for: Progressive Acceleration of Insulin Exposure Over 7 Days of Infusion Set Wear
Source: Diabetes Technol Ther. 2023 Jan 27;25(2):143–7. doi: 10.1089/dia.2022.0323 (PMC9894594; doi:10.1089/dia.2022.0323)
Supplement: Supplemental data [file Supp_FigS2.docx]

**
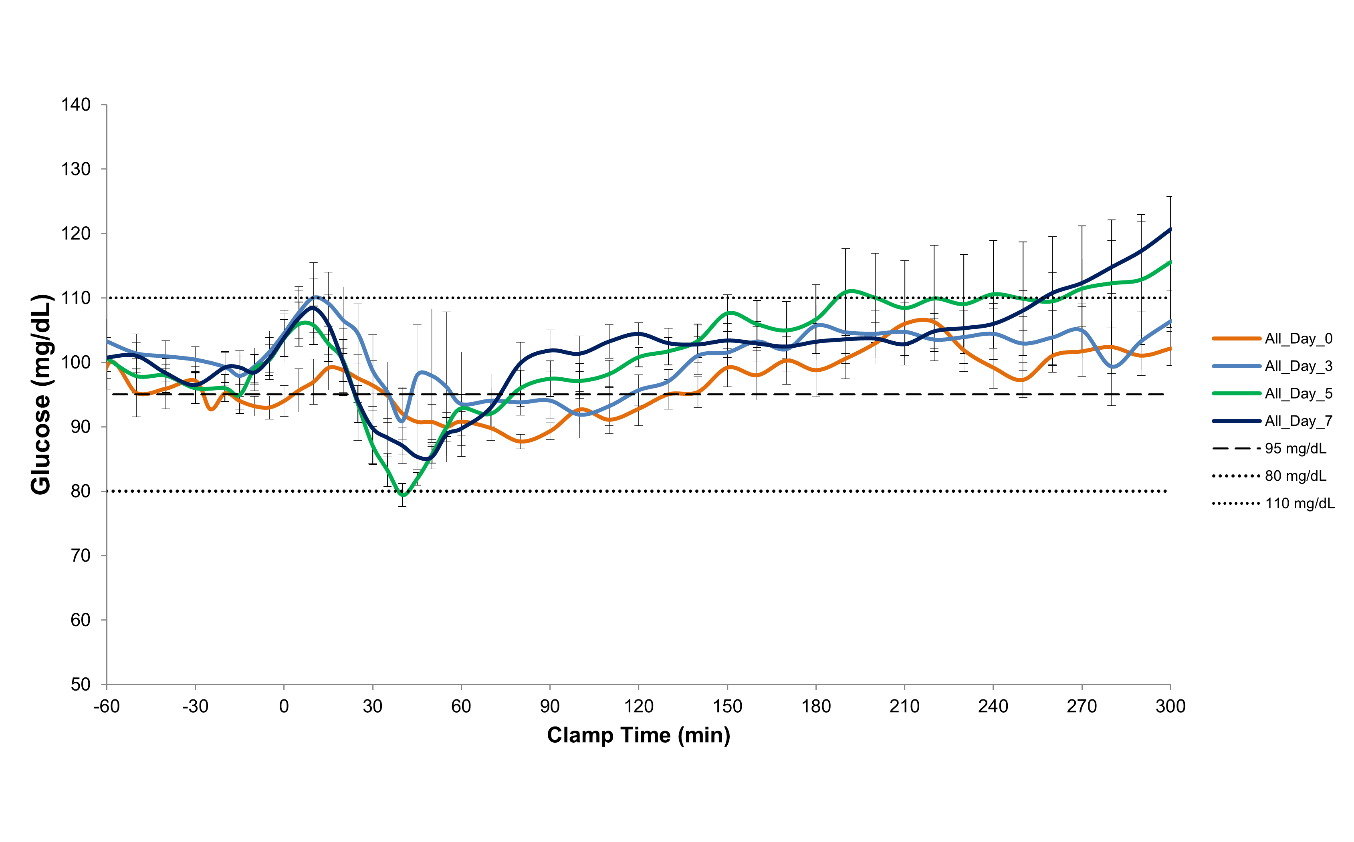
**

**Figure S2:** Blood glucose (BG) concentration of combined data from both treatment groups (CBX and Control IIS) for each clamp day. Curves show average BG concentrations with SEM bars. Bolus was administered at t=0 min.
