# Supplementary material for: Progressive Acceleration of Insulin Exposure Over 7 Days of Infusion Set Wear
Source: Diabetes Technol Ther. 2023 Jan 27;25(2):143–7. doi: 10.1089/dia.2022.0323 (PMC9894594; doi:10.1089/dia.2022.0323)
Supplement: Supplemental data [file Supp_FigS3.docx]

**
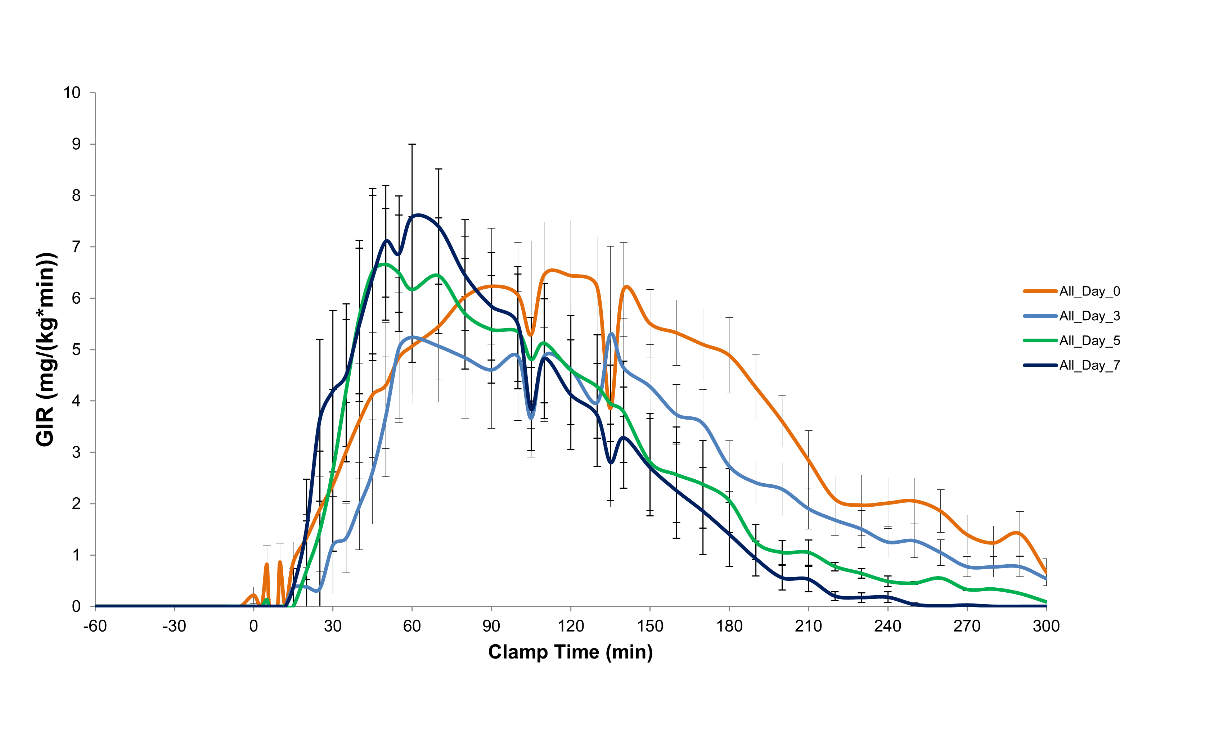
**

**Figure S3:** Glucose infusion rate (GIR) of combined data from both treatment groups (CBX and Control IIS) for each clamp day. Curves show average GIRs with SEM bars. Bolus was administered at t=0 min.
