# Supplementary material for: Progressive Acceleration of Insulin Exposure Over 7 Days of Infusion Set Wear
Source: Diabetes Technol Ther. 2023 Jan 27;25(2):143–7. doi: 10.1089/dia.2022.0323 (PMC9894594; doi:10.1089/dia.2022.0323)
Supplement: Supplemental data [file Supp_FigS4.docx]

**
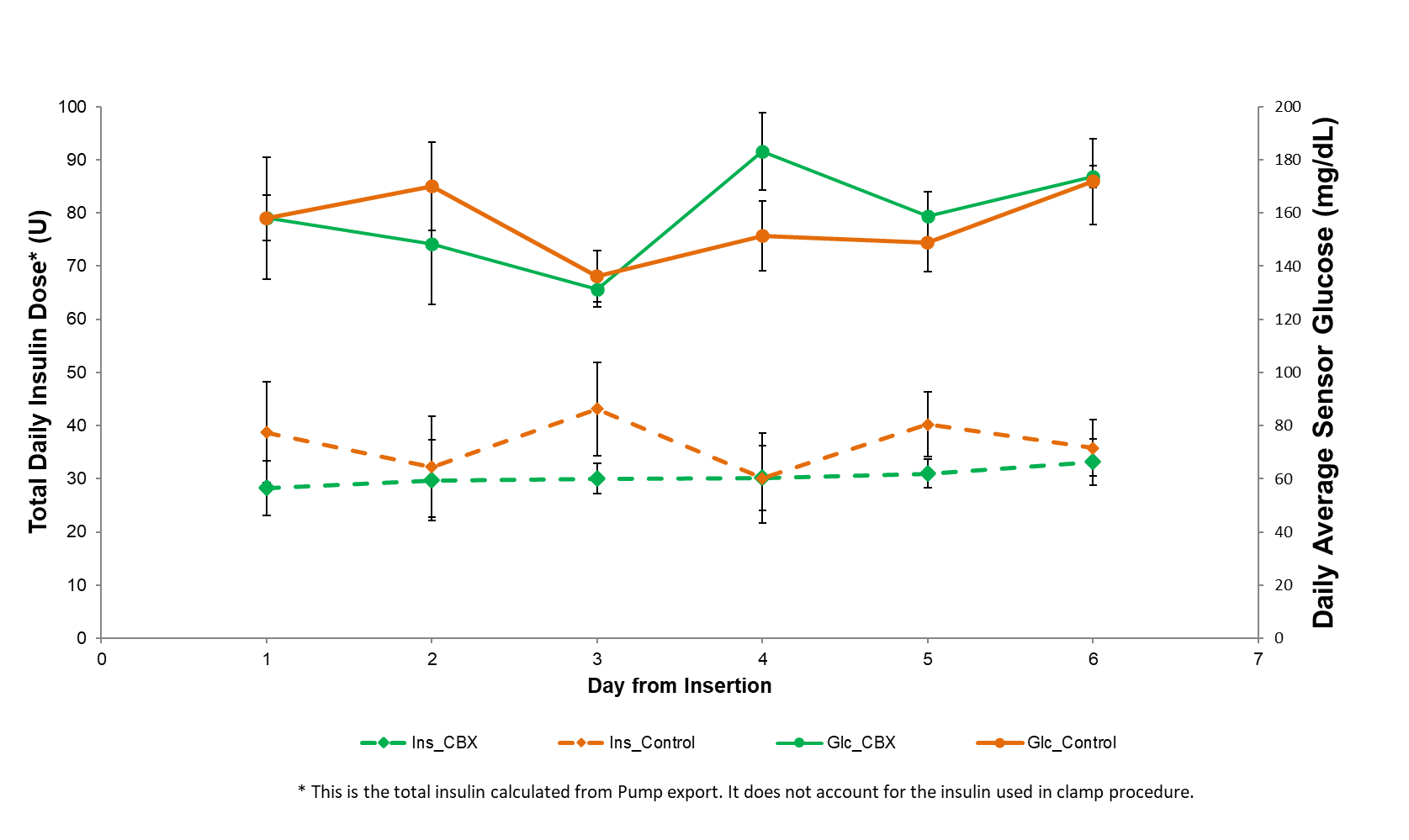
**

**Figure S4:** Average daily sensor glucose and total daily insulin dose (clamps not accounted for) calculated over 24 hour-increments starting at midnight after infusion set insertion. Error bars show SEM. (Ins=Insulin, Glc=Glucose)
