# Supplementary material for: Progressive Acceleration of Insulin Exposure Over 7 Days of Infusion Set Wear
Source: Diabetes Technol Ther. 2023 Jan 27;25(2):143–7. doi: 10.1089/dia.2022.0323 (PMC9894594; doi:10.1089/dia.2022.0323)
Supplement: Supplemental data [file Supp_FigS5.docx]

**
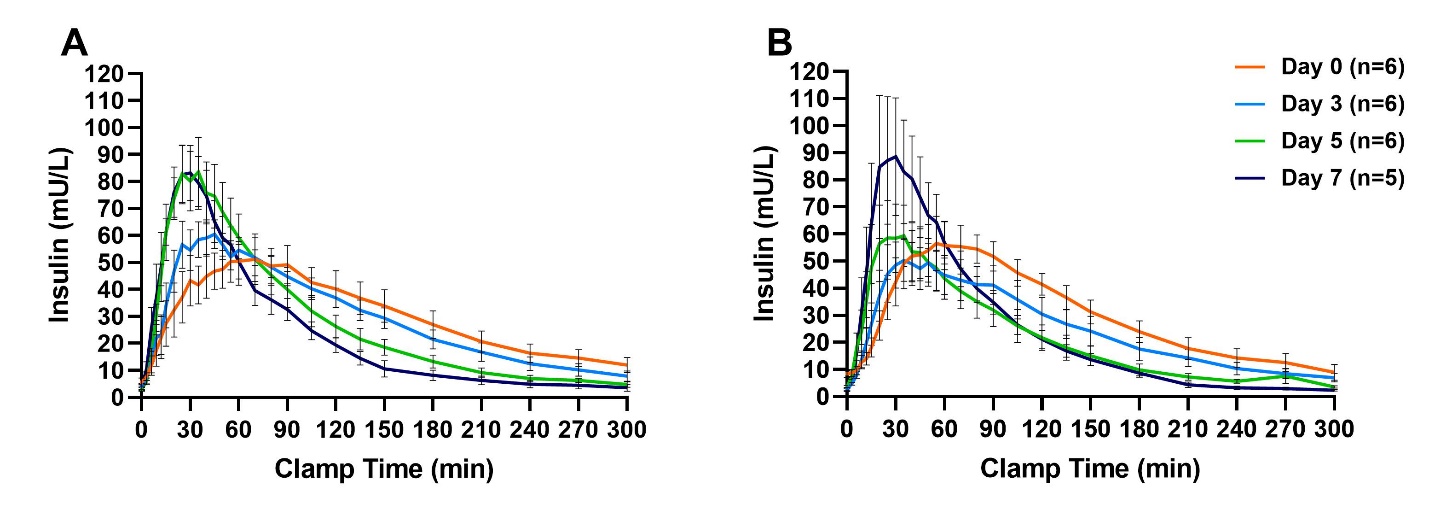
**

**Figure S5:** Insulin exposure over clamp time for **(A)** CBX and **(B)** Control insulin infusion set. Curves show average insulin concentration with SEM bars. Bolus was administered at t=0 min.
