# Supplementary material for: Progressive Acceleration of Insulin Exposure Over 7 Days of Infusion Set Wear
Source: Diabetes Technol Ther. 2023 Jan 27;25(2):143–7. doi: 10.1089/dia.2022.0323 (PMC9894594; doi:10.1089/dia.2022.0323)
Supplement: Supplemental data [file Supp_TableS1.docx]

**Table S1:** Summary of all reported device events, including both protocol-defined failure and other deficiencies.

| **Category** | **CBX** | **Control** |
| --- | --- | --- |
| Total # of Insertions | 6 | 7 |
| Overall | 10 (166.7%) | 6 (85.7%) |
| - Unexplained hyperglycemia (glucose >14 mmol/L) not responsive to a correction bolus* | 1 (16.7%) | 1 (14.3%) |
| - Hyperglycemic with concurrent ketone level >=0.6 mmol/L | 0 (0.0%) | 0 (0.0%) |
| - Infection | 0 (0.0%) | 0 (0.0%) |
| - Occlusion Alarm* | 1 (16.7%) | 0 (0.0%) |
| - Separation Problem | 0 (0.0%) | 1 (14.3%) |
| --- [Adhesive failure] | 0 (0.0%) | 1 (14.3%) |
| - Minor Infections | 5 (83.3%) | 4 (57.1%) |
| --- [Infusion site erythema] | 1 (16.7%) | 1 (14.3%) |
| --- [Infusion site induration] | 1 (16.7%) | 0 (0.0%) |
| --- [Infusion site edema] | 1 (16.7%) | 1 (14.3%) |
| --- [Infusion site pain] | 2 (33.3%) | 1 (14.3%) |
| --- [Purulent discharge] | 0 (0.0%) | 1 (14.3%) |
| - Inability to pierce skin | 3 (50.0%) | 0 (0.0%) |

**event lead to removal of infusion set*
